# First complete genome sequence of a European non-pathogenic rabbit calicivirus (lagovirus GI.3)

**DOI:** 10.1007/s00705-018-3901-z

**Published:** 2018-07-05

**Authors:** Evelyne Lemaitre, Françoise Zwingelstein, Stéphane Marchandeau, Ghislaine Le Gall-Reculé

**Affiliations:** 1 Ploufragan-Plouzané Laboratory, Avian and Rabbit Virology, Immunology and Parasitology Unit, Anses, French Agency for Food, Environmental and Occupational Health and Safety, BP 53, 22440 Ploufragan, France; 2 University Bretagne Loire, CS 54417, 35044 Rennes Cedex, France; 3 Research and Expertise Department, UPFS, ONCFS, National Hunting and Wildlife Agency, CS 42355, 44323 Nantes Cedex 3, France

## Abstract

We report the full genome sequence of the non-pathogenic rabbit lagovirus Lagovirus europaeus/GI.3/*O cun*/FR/2006/06-11 (GI.3/06-11), collected from a healthy French domestic rabbit in 2006, and initially described as 06-11 strain. The sequence reveals a genomic organization similar to lagoviruses. It was 7,436 bases long and contained two open reading frames (ORF). A dipeptide variation at the potential p23/2C-like helicase cleavage site (EE instead of ED) was observed, a feature only shared with non-recombinant pathogenic lagoviruses in GI.2 and with two European brown hare syndrome viruses (EBHSV) collected in 1982 in Sweden. GI.3/06-11 has only one initiation codon at the beginning of the ORF2 like the avirulent Italian rabbit calicivirus (RCV) and EBHSV. Previous genetic analyses based on the capsid gene sequences showed that GI.3/06-11 was closer to the RCV and pathogenic lagoviruses GI.1 strains than other lagoviruses. This study, by revealing that GI.3/06-11 genome sequence significantly clustered with pathogenic GI.2 strains, gives prominence of new genetic relationship among lagoviruses and should contribute to understand the emergence of pathogenic strains.

Lagoviruses (classified within the *Caliciviridae* family) infect Leporid species, hares (*Lepus* sp.) and European rabbits (*Oryctolagus cuniculus*). They are non-enveloped, single-stranded, positive-sense, polyadenylated RNA viruses.

A new nomenclature based on the phylogenetic relationships of the full-length *VP60 capsid* gene has recently been proposed [1]. Taxonomically lagoviruses belong to one single species, but isolates can be divided into two genogroups (GI, GII). Among the lagoviruses infecting rabbits (GI), several genotypes have been described. GI.1 (rabbit hemorrhagic disease virus, RHDV) is responsible for high mortality in domestic and wild rabbit *Oryctolagus cuniculus*. First described in China in 1984, RHDV has become endemic in Europe and Australasia (reviewed in [2]). In 2010, a new genotype of pathogenic lagoviruses (GI.2, RHDV2) emerged in France before spreading in Europe and others countries throughout the world (reviewed in [3]). Several non-pathogenic or mildly pathogenic rabbit lagoviruses phylogenetically distinct from pathogenic forms have also been described [4–8] and are distributed into two other distinct genotypes GI.3 (European rabbit calicivirus, RCV-E1 including the 06-11 strain [6]) and GI.4 (Australian RCV-A1 and European RCV-E2). Among the non-pathogenic lagoviruses a gradient of cross-protection with GI.1 has been demonstrated, and ranges from no cross-protection (GI.3/06-11 [6]), to slight protection (G1.4/RCV-A1 [9]) and full protection (RCV [5]) have been demonstrated. Although divergent from all other lagoviruses, both the Ashington virus [10], RVC and Michigan rabbit calicivirus (MRCV) [4] are unclassified due to the lack of sequence from at least three independent strains. All these data highlights the extent of diversity within viruses classified in the *Lagovirus* genus.

Molecular information on the whole genome of rabbit lagoviruses is essential to better understand the genetic relationships between the different members of this genus and the emergence of pathogenicity [7, 11]. In addition, recombination events within G1.1, G1.2 and G1.4 genomes occur with a relatively high frequency and are likely a major driver of lagovirus evolution [12–15]. Thus, obtaining full-length genomic sequences from divergent lagoviruses should help studies focused on determining recombination events and understanding the evolutionary history of lagoviruses. This is why we undertook to determine the complete sequence of the lagovirus europaeus/GI.3/*O cun*/FR/2006/06-11 strain, named GI.3/06-11 in the manuscript.

GI.3/06-11 was characterized in 2006 in France in a healthy rabbit reared in a rabbitry showing good sanitary conditions [6]. For the full-length genome sequencing, several overlapping PCRs were used to amplify cDNA derived from the viral RNA genome using newly designed primers (primer sequences available upon request) and other primers published for the amplification of the G1.4/MIC-07 genome [8]. PCRs were performed using Expand High Fidelity enzyme (Roche-Applied-Science). Products were analysed by electrophoresis and purified using the MinElute^TM^ PCR Purification Kit (Qiagen). DNA sequences were determined from both strands of the DNA template using the PCR primers and Big Dye Terminator v3.1 (Life Technologies) as recommended by the manufacturer. The sequences were then analyzed with an ABI Prism 3100 Genetic Analyzer (Applied Biosystems). Sequences of the genomic extremities were determined using 5’ and 3’ RACE methods and AmpliTaq Gold Polymerase (Applied Biosystems). To determine the extreme sequence at the 5’ end the viral RNA was reverse transcribed using a gene specific antisense primer (5’ CAACGTCAACAAACTTGTCC 3’ (1L)) and SuperScript^TM^ Reverse Transcriptase (Invitrogen). RNA was then degraded using RNaseH (Invitrogen) and cDNA purified using NucleoSpin® Gel and PCR Clean-up (Macherey-Nagel). A run of C nucleotides were added to the 5’ end using a terminal transferase enzyme (TdT) (Promega). This tailed cDNA was amplified by PCR with an oligo-dG adapter forward primer (5’ GCATCTCGAGGCTTGTGGCGGGGGGH 3’) and the 1L primer, followed by a second PCR with primer (5’ GCATCTCGAGGCTTGTGGC 3’ (19MER)) and the gene specific antisense primer (5’ GATCCAGGAAGACTGGCCAAGCC 3’) as described by [16]. For the 3’ end, cDNA was obtained using an oligo-dT adapter primer (5’ GCATCTCGAGGCTTGTGGCTTTTTTTTTTTTTTTTTTTTTV 3’) and SuperScript^TM^ Reverse Transcriptase. The resulting cDNA was then amplified by PCR with primer 19MER and the gene specific forward primer (5’ GGTTTTAATCCTAATGAAGTTAAG 3’). PCR products were purified and sequenced as described above. Sequence was compiled using Vector NTI Advance 11.5 (Invitrogen). Sequence alignments and pairwise nucleotide or protein distance comparisons (p-distance model) were computed using MEGA software version 7 [17], as well as phylogenetic analyses performed with GI.3/06-11 and rabbit lagovirus complete genomic or coding sequences available in GenBank (http://www.ncbi.nlm.nih.gov/GenBank/index.htlm) including 102 GI.1, 9 GI.2, 45 GI.4, 4 GII.1 and the MRCV sequences. Regarding the GI.2 strains, we exclusively selected the non-recombinant sequences, among them two French sequences recently available (GenBank accession number HE800531/10-28 and HE800532/10-32) for the analyses. For the phylogenetic analyses, the neighbor-joining method (based on the Kimura 2-parameter model) and the maximum likelihood method (based on the General Time Reversible model, uniform rates) were implemented. For the two methods codon positions included were 1st+2nd+3rd.

The full-length genomic sequence (EMBL/Genbank accession number: AM268419) revealed an overall genomic organization consistent with that of the lagovirus genome (Fig. 1) [18]. Without the poly(A) tail but including the 5’ and 3’ non translated regions (NTR), the RNA genome was 7436 nucleotides (nt) long and was close in length to GI.1 (7437 nt). The average genomic nucleotide identity was 86 % with GI.1, 91 % with GI.2, 79 % with GI.4/RCV-A1, 81 % with MRCV and 71 % with GII.1. When compared with other GI viruses for which complete genomic sequences are available, the 5’ NTR was identical in length and nucleotide sequence (except one nt for some GI.1 viruses). The 3’ NTR was 64 nt long and was comparable in length with that of MRCV (65 nt) but slightly longer than GI.1 or GI.4/RCV-A1 (59 and 56 nt, respectively), while slightly shorter than GI.2 (69-70 nt). The length of the two extremities is consistent with the size described for caliciviruses [19]. The genome was organized into two major open reading frames (ORFs). ORF1 was 7029 nt long coding for a 2342*-*amino acid (aa) long polyprotein and was shorter than the corresponding GI.1 or GI.2 ORF1s (2344 aa). This shorter length was due to the deletion of two consecutive codons within the *capsid* gene [6] and not upstream in the genome as described for MRCV or G1.4/RCV-A1. ORF1 has conserved aa sequence motifs typical of helicase, protease and RNA-dependent RNA polymerase (RdRp) nonstructural proteins. These proteins, with the major capsid protein VP60, were in the same order and location as expected for viruses classified within the *Lagovirus* genus. The cleavage sites previously described for the GI.1 polyprotein proteolytic processing [18] were also present. However, a change in the potential dipeptide p23/2C-like helicase cleavage site (E^367^/E^368^ instead of E/D) was observed, a feature only shared with non-recombinant GI.2 from France (10-28 and 10-32 strains) and the Iberian Peninsula [15, 20] and two GII.1 collected in Sweden in 1982 [21]. The average amino acid identity was 95 % with GI.1, 96 % with GI.2, 89 % with GI.4/RCV-A1, 90 % with MRCV and 78 % with GII.1. Out of four sites suggested to differentiate the non-structural proteins of non-pathogenic lagoviruses [7], only three of them were detected in the GI.3/06-11 sequence (aa position 142, 773 and 1659). ORF2 was 342 nt long and coded for a 113-aa long protein corresponding to the minor capsid protein VP10 (or VP2). ORF2 overlapped ORF1 by eight nucleotides and was shorter than that of GI.1, GI.2 or GI.4/RCV-A1 (117 aa). Indeed, whereas GI.1, GI.2 or GI.4/RCV-A1 has two AUG codons in frame that could be responsible for initiation of VP10 translation (located at positions -17 to -15 or -5 to -3, respectively, with regard to the ORF1 termination codon), GI.3/06-11 has only the second one. This characteristic is also shared by RCV, MRCV and GII.1 viruses. ORF2 showed several conserved aa motifs with the lagoviruses minor capsid protein with the average aa identity being 95 % with GI.1, 87 % with GI.2, 86 % with GI.4/RCV-A1, 89 % with MRCV and 69 % with GII.1.

**Fig. 1:**
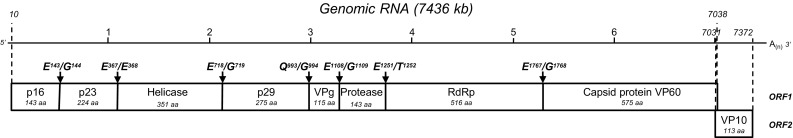
Schematic representation of the RNA genome organization of GI.3/06-11. The expected cleavage sites of the viral protease within ORF1, the name and the size (in amino acids) of the cleavage products are indicated

Irrespective of the method used, phylogenetic analysis of the complete genome sequence did not confirm that which has been previously reported based on analysis of VP60 sequences, i.e. a closer relationship with the RCV and Ashington strains [6]. In contrast, our analysis suggested a closer relationship with GI.2 (Neighbor joining tree, Fig 2). Further molecular analyses, including the search for recombination, that take into account this new complete genome sequence data should be undertaken to better understand the phylogenetic relationships between lagoviruses and the emergence of new pathogenic forms, such as GI.2.

**Fig. 2:**
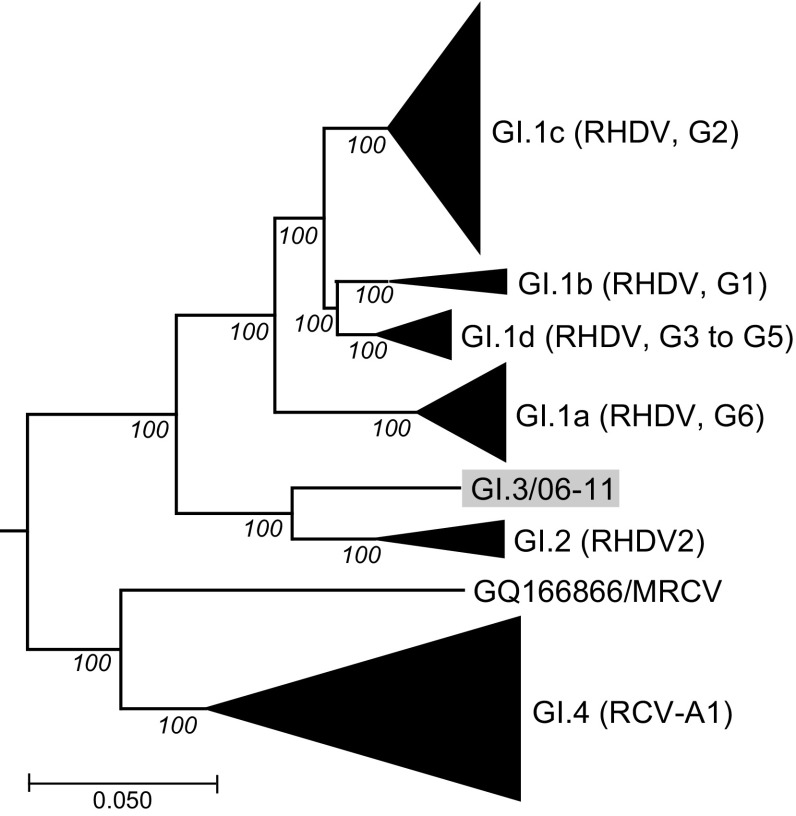
Neighbor Joining phylogenetic tree of lagoviruses (n = 160) and the GI.3/06-11 complete genomic sequences. The lagovirus GII.1/GD89 (GenBank accession number Z69620) was used as an outgroup to root the tree. The scale bar is proportional to the number of nucleotide substitution per site. Bootstrap values for 1,000 replicates are shown before each major branch node. The GI.1, GI.2 and GI.4 branches are collapsed to highlight the main genotypes
